# Mortality Risk Prediction Dynamics After Heart Failure Treatment Optimization: Repeat Risk Assessment Using Online Risk Calculators

**DOI:** 10.3389/fcvm.2022.836451

**Published:** 2022-04-12

**Authors:** Pau Codina, Elisabet Zamora, Wayne C. Levy, Elena Revuelta-López, Andrea Borrellas, Giosafat Spitaleri, Germán Cediel, María Ruiz-Cueto, Elena Cañedo, Evelyn Santiago-Vacas, Mar Domingo, David Buchaca, Isaac Subirana, Javier Santesmases, Rafael de la Espriella, Julio Nuñez, Josep Lupón, Antoni Bayes-Genis

**Affiliations:** ^1^Heart Failure Clinic and Cardiology Service, University Hospital Germans Trias i Pujol, Badalona, Spain; ^2^Department of Medicine, Universitat Autonoma de Barcelona, Barcelona, Spain; ^3^Centro de Investigación Biomédica en Red Enfermedades Cardiovasculares (CIBERCV), Instituto de Salud Carlos III, Madrid, Spain; ^4^UW Medicine Heart Institute, University of Washington, Seattle, WA, United States; ^5^Heart Failure and Cardiac Regeneration (ICREC) Research Program, Health Science Research Institute Germans Trias i Pujol (IGTP), Badalona, Spain; ^6^Department of Medicine, Universitat de Barcelona, Barcelona, Spain; ^7^Barcelona Supercomputing Center, Barcelona, Spain; ^8^Hospital del Mar Medical Research Institute (IMIM), Barcelona, Spain; ^9^Cardiology Department, Hospital Clínico Universitario, Fundación para la Investigación del Hospital Clínico de la Comunidad Valenciana (INCLIVA), Valencia, Spain; ^10^Department of Medicine, Universidad de Valencia, Valencia, Spain

**Keywords:** heart failure, mortality, risk models, risk prediction, prognosis

## Abstract

**Objectives:**

Heart failure (HF) management has significantly improved over the past two decades, leading to better survival. This study aimed to assess changes in predicted mortality risk after 12 months of management in a multidisciplinary HF clinic.

**Materials and Methods:**

Out of 1,032 consecutive HF outpatients admitted from March-2012 to November-2018, 357 completed the 12-months follow-up and had N-terminal pro-B-type natriuretic peptide (NTproBNP), high sensitivity troponin T (hs-TnT), and interleukin-1 receptor-like-1 (known as ST2) measurements available both at baseline and follow-up. Three contemporary risk scores were used: MAGGIC-HF, Seattle HF Model (SHFM), and the Barcelona Bio-HF (BCN Bio-HF) calculator, which incorporates the three above mentioned biomarkers. The predicted risk of all-cause death at 1 and 3 years was calculated at baseline and re-evaluated after 12 months.

**Results:**

A significant decline in predicted 1-and 3-year mortality risk was observed at 12 months: MAGGIC ~16%, SHFM ~22% and BCN Bio-HF ~15%. In the HF with reduced ejection fraction (HFrEF) subgroup guideline-directed medical therapy led to a complete normalization of left ventricular ejection fraction (≥50%) in almost a third of the patients and to a partial normalization (41–49%) in 30% of them. Repeated risk assessment after 12 months with SHFM and BCN Bio-HF provided adequate discrimination for all-cause 3-year mortality (C-Index: MAGGIC-HF 0.762, SHFM 0.781 and BCN Bio-HF 0.791).

**Conclusion:**

Mortality risk declines in patients with HF managed for 12 months in a multidisciplinary HF clinic. Repeating the mortality risk assessment after optimizing the HF treatment is recommended, particularly in the HFrEF subgroup.

## Introduction

Contemporary management of heart failure (HF) has significantly improved over the past two decades, leading to better prognosis (1). Periodic re-evaluation of the risk of death from HF, which may fluctuate, especially in the first few years of the disease, has become increasingly important for optimal patient care. The addition of biomarkers to clinical scores better reflects the pathophysiological pathways in HF and may improve the detection of changes in mortality risk over time. Consistent evidence has linked N-terminal pro B-type natriuretic peptide (NTproBNP), a marker of myocardial stretch, to increased risk of all-cause mortality in patients with HF (2, 3). High sensitivity troponin T (hs-TnT) is a marker of myocyte injury and a strong and independent predictor of all-cause and cardiovascular mortality in HF (4). Finally, interleukin-1 receptor-like 1, known as ST2 (5), reflects myocardial fibrosis and remodeling and has been strongly associated with worsening left ventricular ejection fraction (LVEF) over time (6). Despite the development of several prognostic risk models for HF in the past few years, only some have been externally validated and few include cardiac biomarkers.

Risk prediction models are used in HF to aid clinicians in assessing patient prognosis. Ultimately, they improve the appropriateness and timing of disease-modifying treatments. Previous studies have mainly focused on a single initial risk evaluation, but HF is a non-stable disease. During the first year of HF management, major medication/device changes occur, which lead to substantial alterations in LVEF, functional class, diuretic dose, biomarkers and ultimately life-time survival. Thus, it may be of particular interest to recalculate mortality risk after an initial period of HF management.

The purpose of this study is to asses changes in the predicted mortality risk after a 12-month management period in a multidisciplinary HF unit.

We used three contemporary web-based risk scores: Meta-Analysis Global Group in Chronic HF (MAGGIC-HF) (7) (http://www.heartfailurerisk.org/) and the Seattle HF Model (SHFM) (8) (https://depts.washington.edu/shfm), which include clinical variables, treatments, and blood tests, and version 2.0 of the Barcelona Bio-HF Risk Calculator (BCN Bio-HF) (9, 10) (http://ww2.bcnbiohfcalculator.org), which also includes NTproBNP, hs-TnT, and ST2.

## Materials and Methods

### Study Population and Follow-Up

All consecutive ambulatory patients with HF of different etiologies who were admitted to a structured multidisciplinary HF clinic at a University Hospital between March 2012 and November 2018 were eligible for this study. Patients who completed a 1-year follow-up and had NTproBNP, hs-TnT, and ST2 measurements available at baseline and 12 months were included in the study. Baseline information was obtained at the first visit in the outpatient HF Unit. Patients were referred to the HF clinic mostly by cardiology or internal medicine departments, and to a lesser extent by emergency or other hospital departments. The criteria for referral to the clinic were HF according to the ESC definition, with at least one hospitalization and/or reduced systolic function, as described previously (11). For follow-up, all patients regularly visited the HF clinic and were treated according to a unified protocol. Follow-up visits comprised a minimum of quarterly visits with a nurse, one visit with a physician (cardiologist, internist, or family physician) every 6 months, and optional visits with specialists in geriatrics, psychiatry, rehabilitation, endocrinology, or nephrology.

During the baseline visit, patients provided written consent for the use of their clinical data for research purposes. Demographic, clinical, echocardiographic, and analytical data were recorded in the REGI-UNIC database. Data that were not routinely recorded in that database were obtained by reviewing electronic patient health records.

### Outcomes

Change in the risk of all-cause death was the main endpoint for comparing the different risk calculators. Risk of all-cause death at 1 and 3 years was calculated at baseline and re-evaluated after a 12-month follow-up period. Follow-up was closed on 30 September 2021. Fatal events were identified by reviewing the patient health records from hospital wards, the emergency room, and general practitioners or by contacting their relatives. Data were verified with the databases of the Catalan and Spanish Health Systems and the Spanish National Death Index (INDEF).

The study was performed in compliance with the laws that protect personal data and the international guidelines on clinical investigations from the World Medical Association's Declaration of Helsinki. The local ethics committee approved the study.

### Biomarker Assays

All samples were obtained between 9:00 am and 12:00 pm. The three biomarkers were analyzed from the same blood sample: NTproBNP from a fresh plasma sample and hs-TnT and ST2 from serum stored at −80°C without previous freeze-thaw cycles. NTproBNP levels were determined by an immuno-electrochemiluminescence assay on the Modular Analytics E 170 instrument (Roche Diagnostics, Switzerland). This assay had <0.001% cross-reactivity with bioactive BNP. The assay had inter-run coefficients of variation ranging from 0.9 to 5.5%. Since 2016, NTproBNP and hs-TnT have been determined by electrochemiluminescence immunoassays on a Cobas E601 platform (Roche Diagnostics, Switzerland). ST2 was measured by immunoturbidimetry using the SEQUENT-IA reagent kit (Critical Diagnostics, Ireland) and an AU-5800 platform (Bekman Coulter, Ireland).

### Statistical Analysis

Categorical variables were expressed as absolute numbers and percentages. Continuous variables were expressed as the mean ± standard deviation (SD) or the median and interquartile range (IQR [Q1–Q3]) according to normal or non-normal data distributions. Normal distributions were assessed with normal Q–Q plots. Between-group comparisons were performed using McNemar test for paired categorical variables and the paired Student's *t*-test or Mann-Whitney *U* test for continuous variables as appropriate. Missing values were treated by imputing the median values.

Risk of all-cause death at 1 and 3 years year was calculated at baseline with the three online calculators and then re-evaluated after a 12-month follow-up period. The Wilcoxon matched-pairs signed-rank test was used to assess changes in mortality risk due to the much skewed distribution of predicted risks. A meaningful difference in the score was defined as at least a 1% absolute change in the estimated value to enter into the increased or decreased categories. Cohen's kappa coefficient was used to measure inter-score reliability when categorizing patients into the three groups of change in risk of death: one group included patients who presented an increase in mortality risk, another one patients who presented a decrease in mortality risk, and the third patients whose risk did not meaningfully changed.

Statistical analyses were performed using SPSS 24 software (SPSS Inc., Chicago, IL, USA), STATA V.15.1 software (StataCorp, College Station, Texas, USA), and R software (A Language and Environment for Statistical Computing) distributed by the R Core Team (2017; R Foundation for Statistical Computing, Vienna, Austria). A two-sided *p* < 0.05 was considered significant.

## Results

A total of 1,032 consecutive patients were admitted to the HF clinic during the inclusion period. Of these patients, 935 were alive after 1 year and 578 patients were excluded because they lacked an NTproBNP, hs-TnT, or ST2 measurement at baseline or 12 months.

Our final cohort included 357 patients. None of the patients included in this study had participated previously in the BCN Bio-HF derivation cohort. Supplementary Table 1 compares included and excluded patients.

### Clinical and Demographic Characteristics of the Study Population

The patients included were predominantly men, aged 65.2 ± 12.3 years, with reduced LVEF (37.8 ± 13.6%), and mostly classified as NYHA class II (75.4%). Ischaemic heart disease was the most prevalent etiology (37.3%). Contemporary HF treatment was optimized according to international guidelines. Table 1 provides the demographic, clinical, biochemical, and echocardiographic characteristics and treatments of the studied cohort at baseline and after 12 months of follow-up.

**Table 1:** Comparison between population characteristics at baseline and after a 12-month management period.

	**Baseline (*n* = 357)**	**12 months (*n* = 357)**	***p*-value**
Age, years	65.2 ± 12.3	66.2 ± 12.3	–
Male, *n* (%)	255 (71.4)	255 (71.4)	–
BMI (kg/m^2^)	28.4 ± 4.9	28.53 ± 5.7	0.98
Ischemic etiology	133 (37.3)	133 (37.3)	–
Heart failure duration, months	4 (1–24)	16 (13–36)	–
Diabetes	144 (40.3)	144 (40.3)	–
COPD	59 (16.5)	59 (16.5)	–
Smoking	68 (19.0)	68 (19.0)	–
Systolic BP	129.1 ± 21.3	127.1 ± 19.9	0.11
**NYHA functional class**, ***n*** **(%)**
I	43 (12.0)	44 (12.3)	0.89
II	269 (75.4)	275 (77.0)	0.57
III	45 (12.6)	38 (10.6)	0.36
IV	0 (0)	0 (0)	–
LVEF ≤ 40%, *n* (%)	233 (65.2)	107 (30.0)	<0.001
LVEF 41–49%, *n* (%)	62 (17.4)	95 (26.6)	0.003
LVEF ≥ 50%, *n* (%)	62 (17.4)	155 (43.4)	<0.001
**Blood tests**
Hemoglobin, g/dL	13.3 ± 1.8	13.4 ± 2.2	0.18
Sodium, mmol/L	137.5 ± 3.4	139.7 ± 2.9	<0.001
Uric acid, umol/L	433.9 ± 93.9	419.5 ± 112.5	0.033
eGFR, mL/min/1.73 m^2^	65.0 ± 26.5	62.4 ± 26.3	0.010
Total cholesterol, mmol/L	4.23 ± 0.86	4.27 ± 0.89	0.44
NT-proBNP, pg/mL	1,499 [680–3,434]	643 [233–1,933]	<0.001
ST2, ng/ml	21.0 [15.0–30.0]	20.0 [14.0–29.0]	0.23
hs-TnT, pg/ml	26.3 [14.6–42.8]	18.7 [11.2–32.9]	0.002
**Treatments**, ***n*** **(%)**
Beta-blocker	300 (84.0)	324 (90.8)	<0.001
ACEI/ARB	262 (73.3)	233 (65.3)	0.003
ARNI	13 (3.6)	39 (10.9)	<0.001
Loop diuretics			
Furosemide >40 mg/d	188 (52.7)	88 (24.6)	<0.001
Furosemide ≤ 40 mg/d	169 (47.3)	269 (75.4)	<0.001
MRA	65 (18.2)	228 (64.0)	<0.001
CRT	22 (6.2)	33 (9.2)	<0.001
ICD	31 (8.7)	41 (11.5)	<0.001

*Values are the mean ± standard deviation, n (%), or median [interquartile range], as indicated*.

*ACEI, angiotensin-converting enzyme inhibitor; ARB, angiotensin II receptor blocker; ARNI, angiotensin receptor neprilisyn inhibitor; BMI, body mass index; BP, blood pressure; BUN, blood urea nitrogen; COPD, chronic obstructive pulmonary disease; CRT, cardiac resynchronization therapy; eGFR, estimated glomerular filtration rate; ICD, implantable cardioverter-defibrillator; LVEF, left ventricular ejection fraction; MRA, mineralocorticoid receptor antagonist; N/A, not available; NT-proBNP, N-terminal pro-brain natriuretic peptide; NYHA, New York Heart Association; ST2, interleukin 1 receptor-like 1; hs-TnT, high sensitivity troponin T*.

Supplementary Table 2 shows the number and management of missing values in the study cohort. The estimated Kaplan-Meier mortality at 1, 2 and 3 years was 4.2, 8.1 and 15.7%.

### Left Ventricular Ejection Fraction Trajectories

**Figure 1:**
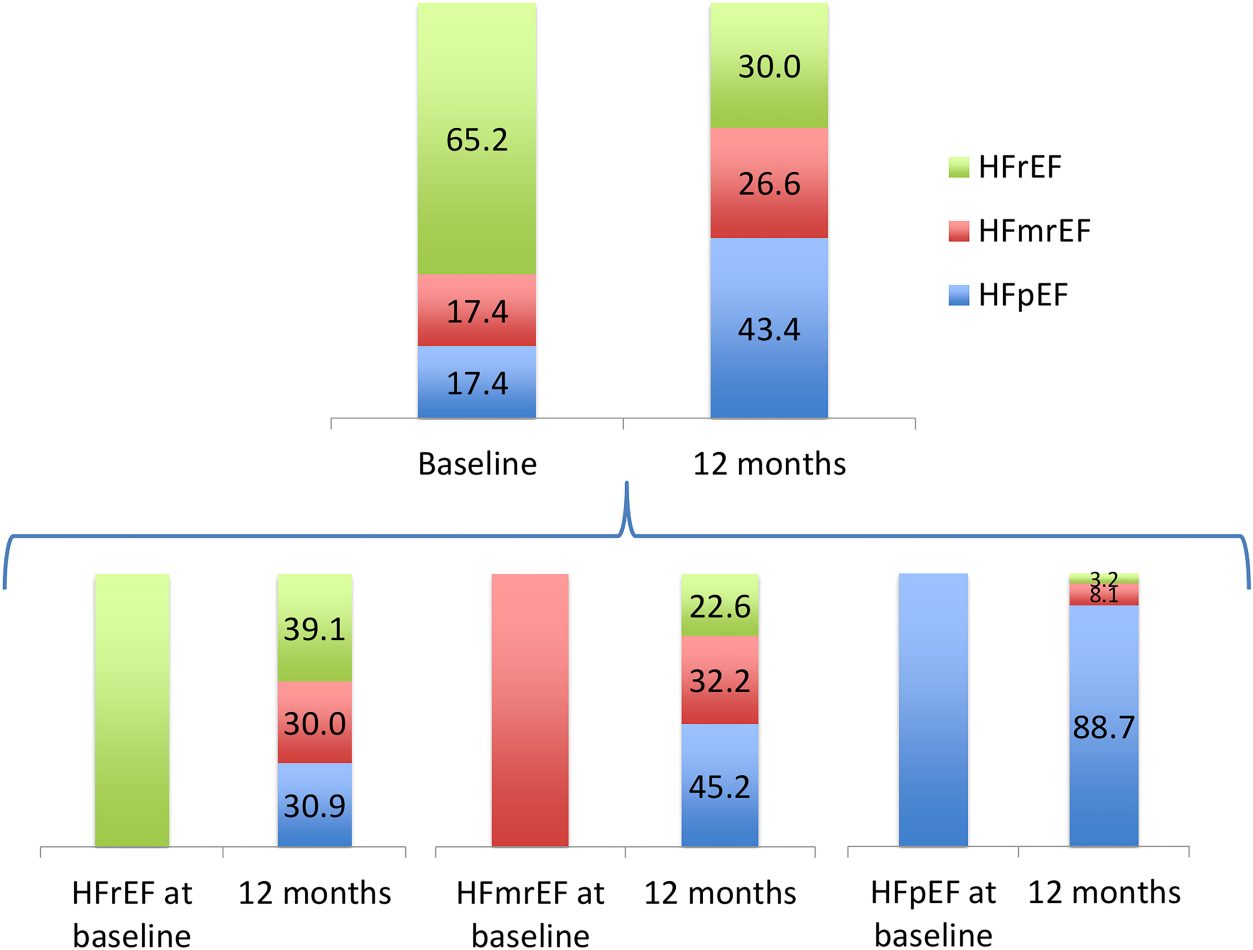
Percentage of heart failure patients with reduced ejection fraction (HFrEF), mildly reduced ejection fracion (HFmrEF) and preserved ejection fraction (HFpEF) at baseline and after 12 months.

There was a marked improvement in LVEF after 1 year. The mean LVEF was 37.8 ± 13.6 at baseline and improved to 47.5 ± 13.2 at 1 year (*p* < 0.001). Figure 1 depicts the percentage of HF patients with reduced ejection fraction (HFrEF), mildly reduced ejection fraction (HFmrEF) and preserved ejection fraction (HFpEF) at baseline and after 12 months.

The relative change in LVEF inversely correlated with changes in the risk of all-cause death estimated by the three calculators (Table 2). This was also accompanied by an improvement in the NYHA functional class.

**Table 2:** Correlation between relative changes in all-cause death risk at 1 year for every calculator and relative changes in LVEF and biomarkers.

	**SHFM**		**MAGGIC-HF**		**BCN-Bio-HF**	
	**rho**	***p*-value**	**rho**	***p*-value**	**rho**	***p*-value**
**LVEF**	−0.13	0.02	−0.52	<0.001	−0.23	<0.001
**NTproBNP**	0.18	<0.001	0.37	<0.001	0.43	<0.001
**Hs-TnT**	0.31	<0.001	0.32	<0.001	0.53	<0.001
**ST2**	0.12	0.02	0.11	0.04	0.37	<0.001

*LVEF, left ventricular ejection fraction; NT-proBNP, N-terminal pro-brain natriuretic peptide; ST2, interleukin 1 receptor-like 1; hs-TnT, high sensitivity troponin T*.

### Biomarkers

**Figure 2:**
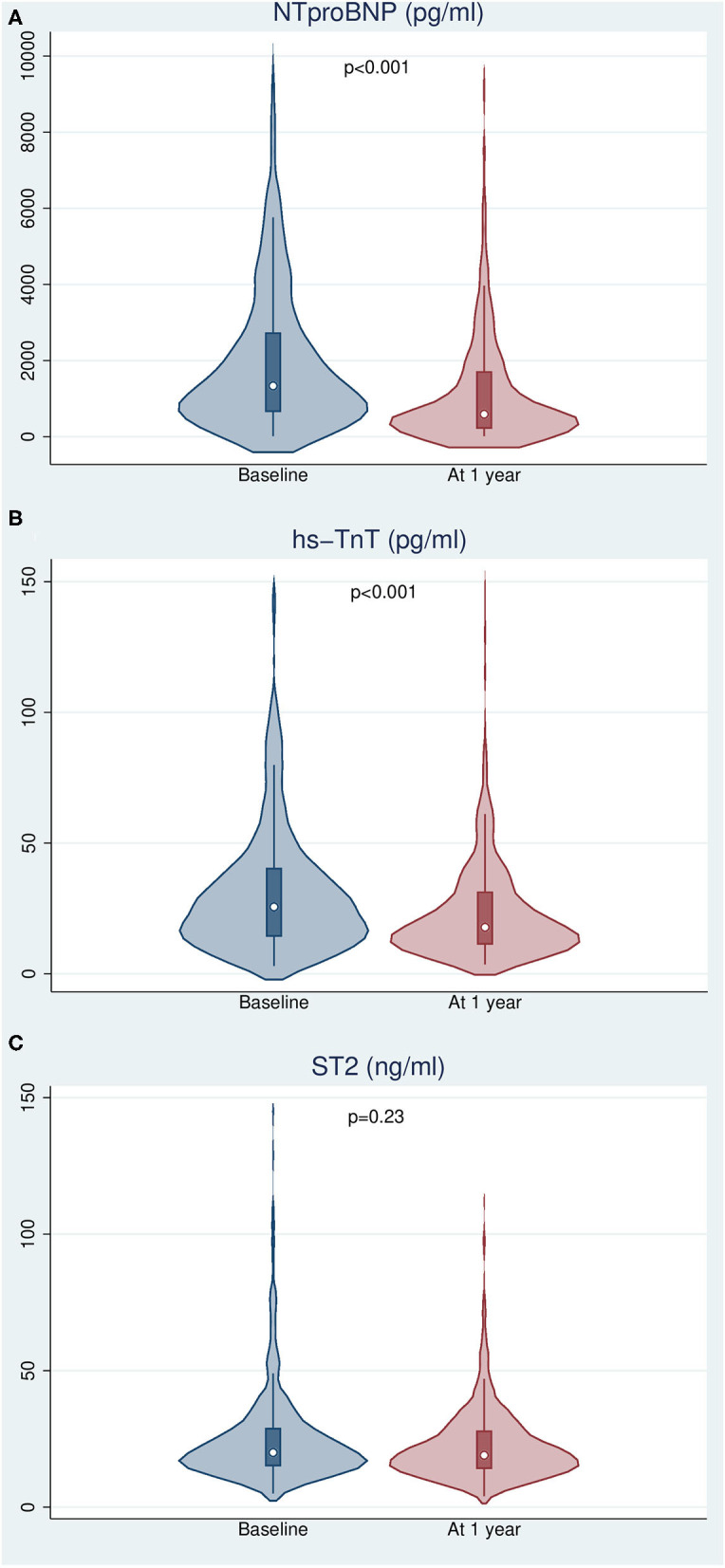
Changes in biomarker levels after 12 months of follow-up. **(A)** N-terminal pro-brain natriuretic peptide. **(B)** High sensitivity troponin T. **(C)** Interleukin 1 receptor-like 1 (ST2).

There was a significant decline in the concentration of NTproBNP and hs-TnT, with median relative reductions of 57.1 and 46.6%, respectively (*p* < 0.001). A modest non-significant 4.8% reduction in the concentration of ST2 (*p* = 0.23) was observed (Figure 2). We found a significant correlation between biomarker dynamics and changes in the estimated risk of all-cause death using the three calculators, including MAGGIC-HF and SHFM, which do not include such biomarkers (Table 2).

### All-Cause Mortality Risk

**Figure 3:**
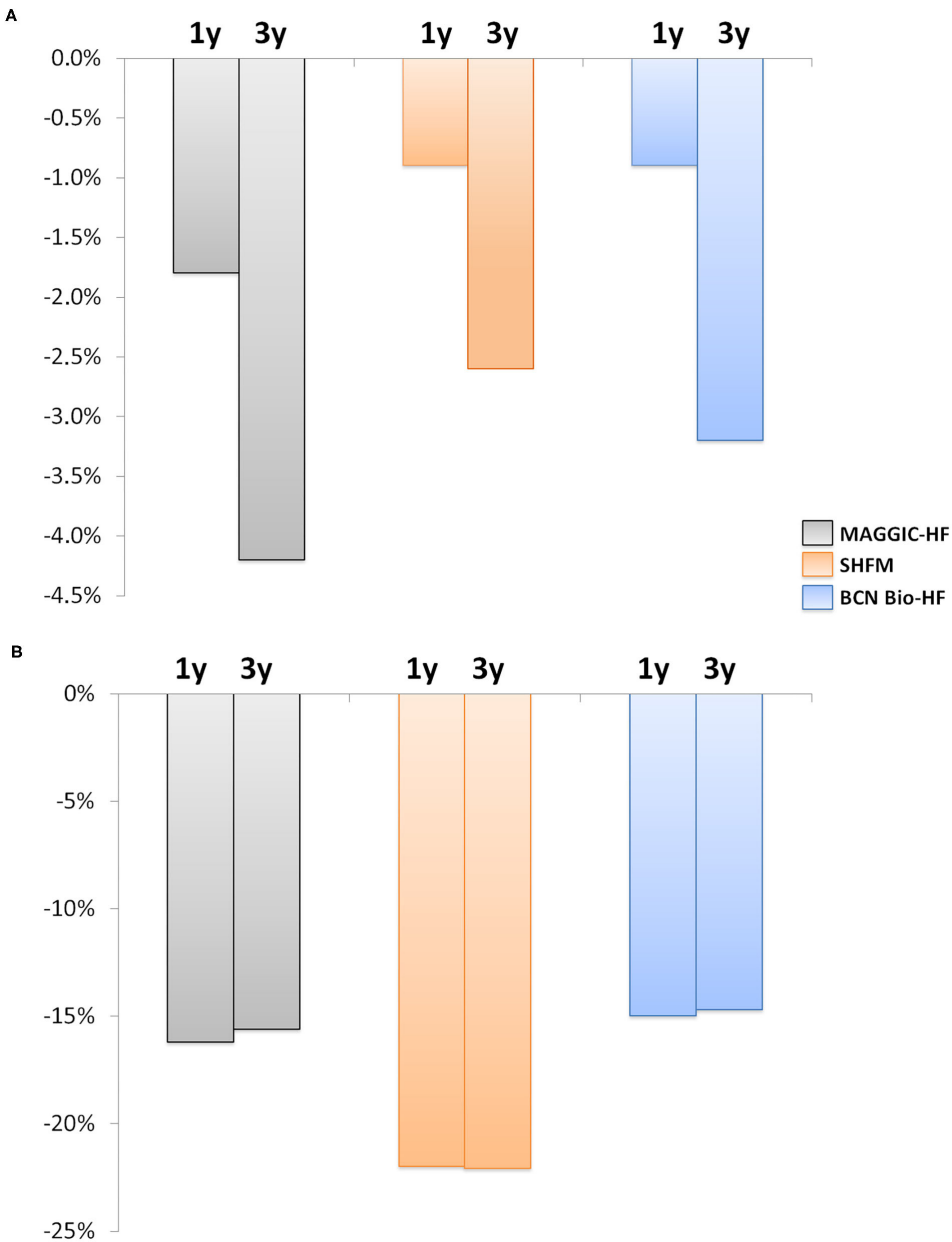
Change in the risk of all-cause death at 1 and 3 years after 12 months of follow-up. **(A)** Absolute risk change. **(B)** Relative risk change. The estimated risk of death decreased significantly with the three calculators (all *p* < 0.001). Gray, MAGGIC; Orange, SHFM; Blue, BCN Bio-HF.

Supplementary Figure 1 depicts 1- and 3-year predicted all-cause risk of death by every calculator, both at baseline and after 12 months of management. The distribution was extremely skewed, so median values were considered for analyses. A significant global reduction in the predicted risk of all-cause mortality was observed with the three risk scores after a 12-month follow-up (Figure 3, Table 3), despite the inherent increase in age and HF duration.

**Table 3:** 1- and 3-year mortality risk estimation by studied HF calculators at baseline and after 12 months of follow-up.

	**Observed^#^**	**SHFM**		**MAGGIC-HF**		**BCN Bio-HF**	
		At baseline^*^	At 12 months^*^	At baseline^*^	At 12 months^*^	At baseline^*^	At 12 months^*^
**1 year**	4.2%	4.1% (2.5–6.8)	3.2% (2.0–4.8)	11.1% (7.0–17.5)	9.3% (5.2–16.0)	6.0% (2.8–13.2)	5.1% (2.0–11.0)
**3 years**	15.7%	12.3% (7.8–19.9)	9.7% (6.3–14.3)	26.9% (17.5–39.7)	22.7% (13.4–36.9)	21.8% (10.6–43.1)	18.6% (7.6–37.2)

*Statistical comparison: p < 0.001 for all risk comparisons*.

* *Median (IQR)*.

# *Kaplan Meyer estimate*.

**Figure 4:**
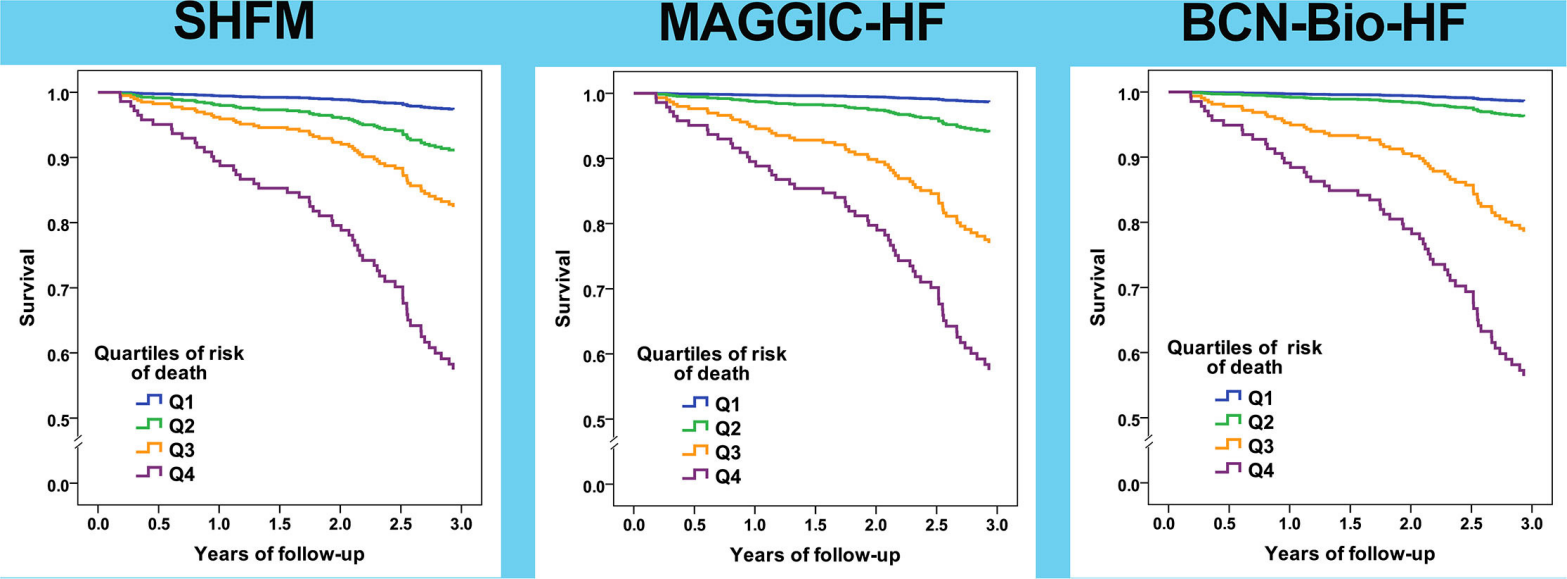
Survival curves based on quartiles of risk of all-cause death for the three calculators based on risk estimated after 12 months of follow-up.

Remarkably, the re-calculated risks after 12 months of HF management allowed an accurate identification of the risk of death (Table 4, Figure 4). Harrell's C statistic for 3-year mortality predictions were 0.762 (95% CI 0.699–0.824), 0.781 (95% CI 0.726–0.836) and 0.791 (95% CI 0.738–0.844) using MAGGIC-HF, SHFM and BCN Bio-HF respectively.

**Table 4:** Cox regression based on quartiles of the risk estimated at 1 year.

	**Risk estimated at 1 year**		
	**HR**	**95% CI**	***p*-value**
**SHFM**
Q1	1		
Q2	3.55	0.74–17.1	0.11
Q3	7.23	1.64–31.8	0.009
Q4	20.7	4.96–86.3	<0.001
**MAGGIC**
Q1	1		
Q2	4.56	0.53–39.1	0.17
Q3	19.3	2.57–145.1	0.004
Q4	40.9	5.59–298.9	<0.001
**BCN Bio-HF**
Q1	1		
Q2	2.72	0.28–26.1	0.38
Q3	17.0	2.26–127.8	0.006
Q4	40.3	5.52–294.3	<0.001

Correlations between the three studied risk scores with regard to the absolute change in risk of all-cause death after a 12-month follow-up were poor (Supplementary Figure 2). Although the majority of patients presented with a reduction in mortality risk using the three calculators after 12 months of HF management, a non-negligible proportion of patients presented with a meaningful increase in risk: 20.2% with SHFM, 23.8% with BCN Bio-HF, and 24.4% with MAGGIC-HF. Supplementary Figure 3 shows correlation between risk estimation at baseline and after 12 months of management. When patients were categorized into three groups according to their change in mortality risk (decrease vs. increase vs. no-change), kappa coefficients between the scores were poor (Table 5).

**Table 5:** Agreement between calculators and Cohen's kappa coefficients regarding increased risk of death after 1 year of follow-up.

	**MAGGIC**	**SHFM &**	**MAGGIC &**
	**& SHFM**	**& BCN Bio-HF**	**& BCN Bio HF**
Agreement (%)	50.2	59.5	50.4
Kappa	0.22	0.37	0.23

## Discussion

In the present study, we aimed to assess changes in the predicted mortality risk after a 12-month management period in a multidisciplinary HF clinic. We used three contemporary web-based risk scores: MAGGIC-HF (6), SHFM (7), and BCN Bio-HF (8, 9).

The most important finding of this study was that despite the inherent increase in age and HF duration, a significant global reduction in the estimated mortality risk occurs with all HF risk scores after a 12-month management period. This reduction in mortality risk reflects the relevance of following guideline recommendations and ensuring that the majority of patients receive evidence-based drugs and cardiac devices when appropriate.

Periodic re-evaluation of the risk of death from HF, which fluctuates in the first few years of the disease, has become increasingly important for optimal patient care. It is vital that patients receive accurate information concerning prognosis in order to make decisions and plans for the future.

Interestingly, the 1- and 3 year recalculated predicted mortality of BCN Bio-HF and SHFM were closer to the observed mortality than the MAGGIC-HF predicted mortality. The recalculated risks after 1 year of HF management better identified the risk of death than the observed change in the risk, suggesting that it is more accurate to consider the last recalculated risk during patient follow-up in order to better tailor therapeutic options. To the best of our knowledge, this study is the first to assess the dynamics of death risk prediction with these contemporary HF risk scores in a real-life prospective cohort of patients managed at a multidisciplinary HF clinic.

There was a marked increase in LVEF at 1 year, which was accompanied with a significant reduction in the concentration of the three studied biomarkers. Recent evidence indicates that HF includes multiple diverging patient-oriented phenotypes, resulting in a broad spectrum of time-dependent LVEF trajectories (12, 13). In the HFrEF subgroup guideline-directed medical therapy led to a complete normalization of LVEF (≥50%) in almost a third of the patients and to a partial normalization (41–49%) in 30% of them. This may explain the lower dose of furosemide needed at 12 months.

On the other hand, only 3.2% of HFpEF patients developed a HFrEF phenotype at the end of the first year. Thus, it might be particularly significant to re-evaluate HF prognostic indicators in the HFrEF subgroup.

Several prognostic risk models of HF have been developed in recent years, but only a few have been externally validated, and even fewer include cardiac biomarkers known to refine death risk prediction in HF patients. A recent head-to-head comparison of contemporary HF risk scores suggested that natriuretic peptides add value to HF risk stratification tools (14). The incorporation of biomarkers in HF scores may not only improve discrimination at baseline, but also reflect changes in mortality risk over time. In the present study, NTproBNP and hs-TNT had a significant reduction in their values, whereas ST2 did not. However, a significant correlation was found between changes observed in the three biomarkers (NTproBNP, hs-TnT, and ST2), together with changes in the LVEF and changes in all-cause death risk assessed by the three calculators. Correlation was higher with the BCN-Bio-HF calculator, likely due to these biomarkers being included as variables in the calculator. Nevertheless, it is remarkable that changes in the three biomarkers also significantly correlated with changes in the estimation of risk by SHFM and MAGGIC-HF at 12 months.

### Study Limitations

Our study has some limitations. First, our analysis was performed only for “completers,” that is, patients with complete 12-months follow-up and with both baseline and 1-year blood samples available. It is not possible to predict the effect that the “non-completers” may have had on some of the analyses. Nevertheless, in the subgroup of patients who died during first year follow-up, the 1-year average mortality risk estimated by MAGGIC, SHFM and BCNBioHF was 26.8, 10.6 and 55.5%, respectively. Second, only BCNBioHF allows estimating HF related hospitalizations, so we could not compare these events beyond all-cause death. Third, although our sample comprised patients with general HF, most patients had depressed LVEF and were treated at a multidisciplinary HF clinic in a tertiary hospital. In addition, most of the patients were referred from the Cardiology Department. Thus, our cohort was mostly comprised of relatively young men with HF with a significant proportion from ischemic etiology. Consequently, our results may not be generalizable to a global HF population that may include patients with HF with preserved ejection fraction. Although patients with more than three missing values were excluded, we could not rule out the possibility of bias due to the missing variables. Our sample is limited and from a single center over a long time period. A more robust comparison of risk scores should be carried out in a larger multi-center contemporary patient population. Although none of the patients included in the present study had participated previously in the BCN Bio-HF derivation cohort, they were derived from the same clinic as the original BCN-Bio-HF calculator, so we cannot discard potential bias in the analysis.

## Conclusion

After a 12-month management period in a multidisciplinary HF clinic, the estimated risk of all-cause-mortality was significantly reduced with three contemporary HF risk scores. Therefore, repeat assessment of all-cause death risk in patients with HF is recommended, particularly in the HFrEF subgroup.

In contemporarily treated HF outpatients, recalculated risk with SHFM and BCN Bio-HF after 12 months of management showed closer results to the observed mortality together with better discrimination.

## Data Availability Statement

The raw data supporting the conclusions of this article will be made available by the authors, without undue reservation.

## Ethics Statement

Ethical review and approval was not required for the study on human participants in accordance with the local legislation and institutional requirements. The patients/participants provided their written informed consent to participate in this study.

## Author Contributions

PC, JL, AB-G, and WL drafted the work. EZ, ER-L, AB, GS, GC, MR-C, EC, ES-V, MD, DB, IS, JS, RE, and JN revised it critically. All authors agree to be accountable for all aspects of the work in ensuring that questions related to the accuracy or integrity of any part of the work are appropriately investigated and resolved and made substantial contributions to the conception of the work and provided approval for publication of the content.

## Funding

The NTproBNP assays were partially provided by Roche Diagnostics and the ST2 assays by Critical Diagnostics. Roche Diagnostics and Critical Diagnostics had no role in the design of the study or the collection, management, analysis, or interpretation of the data.

## Conflict of Interest

AB-G has received lecture honoraria from Abbott, AstraZeneca, Boehringer-Ingelheim, Novartis, Vifor, Roche Diagnostics, and Critical Diagnostics. AB-G and JL report a relationship with Critical Diagnostics. WL—steering committee for Respircardia and Cardiac Dimensions, clinical event committees for Abbott, EBR Systems, Beckman Coulter NTproBNP, and Siemens NTproBNP. He has grant support from Medtronic. He is a consultant to Medtronic and Impulse Dynamics. University of Washington CoMotion holds the copyright to the SHFM. The remaining authors declare that the research was conducted in the absence of any commercial or financial relationships that could be construed as a potential conflict of interest.

## Publisher's Note

All claims expressed in this article are solely those of the authors and do not necessarily represent those of their affiliated organizations, or those of the publisher, the editors and the reviewers. Any product that may be evaluated in this article, or claim that may be made by its manufacturer, is not guaranteed or endorsed by the publisher.
