# Supplementary material for: Mortality Risk Prediction Dynamics After Heart Failure Treatment Optimization: Repeat Risk Assessment Using Online Risk Calculators
Source: Front Cardiovasc Med. 2022 Apr 12;9:836451. doi: 10.3389/fcvm.2022.836451 (PMC9039357; doi:10.3389/fcvm.2022.836451)

Supplementary material

Supplementary Table 1. Comparison between baseline characteristics of included and excluded patients.

	**Included (n=357)**	**Excluded* (n=578)**	**P-value**
Age, years	65.2 ± 12.3	64.8 ± 13.8	0.72
Male, n (%)	255 (71.4)	402 (69.6.2)	0.54
BMI (kg/m^2)	28.4 ± 4.9	27.4± 4.8	0.19
Ischaemic aetiology	133 (37.3)	219 (37.9)	0.85
Heart failure duration, months	4 [1-24]	5 [1-36]	0.21
Hypertension	236 (66.1)	381 (65.9)	0.95
Diabetes	144 (40.3)	270 (46.7)	0.06
COPD	59 (16.5)	79 (13.7)	0.23
Current smoker	68 (19.0)	109 (18.9)	0.94
Systolic BP	129.1 ±21.3	127.7 ±21.4	0.33
NYHA functional class, n (%)			
I	43 (12.0)	58 (10.0)	0.34
II	269 (75.4)	405 (70.0)	0.08
III	45 (12.6)	113 (19.6)	<0.01
IV	0 (0)	2 (0.3)	0.27
Atrial fibrillation/flutter, n (%)	70 (19.6)	125 (21.6)	0.46
LVEF, %	37.8 ± 13.6	38.0± 15.2	0.86
**Blood tests**			
Haemoglobin, g/dL	13.3 ± 1.8	12.8 ± 1.9	<0.01

Lymphocytes, n (%)	22.6 ± 9.1	22.2 ± 8.4	0.50
Sodium, mmol/L	137.5 ± 3.4	137.5 ± 3.4	0.99
Potassium, mmol/L	4.3 ± 0.5	4.3 ± 0.5	0.27
Uric acid, umol/L	439 ± 131	438± 139	0.95
eGFR, mL/min/1.73m^2	65.0 ± 26.5	62.6 ± 29.6	0.22
Total cholesterol, mmol/L	4.26 ± 1.15	4.08 ± 1.17	0.09
NT-proBNP, pg/mL	1499 [680-3434]	1640 [717-4000]	0.06
ST2, ng/ml	36.8 [27.5-50.0]	N/A	
hs-TnT, pg/ml	26.3 [14.6-42.8]	N/A	
**Treatments, n (%)**			
Beta-blocker	300 (84.0)	472 (81.6)	0.35
ACEI/ARB/ARNI	277 (77.5)	397 (68.7)	<0.01
Loop diuretics			
Furosemide >40mg/d	188 (52.7)	314 (54.3)	0.62
Furosemide ≤40mg/d	169 (47.3)	264 (45.7)	0.62
MRA	65 (18.2)	58 (10.0)	<0.01
CRT	22 (6.2)	26 (4.5)	0.26
ICD	31 (8.7)	59 (10.2)	0.44

* In the excluded category, only the patients excluded for reasons other than death were included.

Values are the mean ± standard deviation, n (%), or median [interquartile range], asindicated.

ACEI: angiotensin-converting enzyme inhibitor; ARB: angiotensin II receptor blocker; ARNI: angiotensin receptor neprilisyn inhibitor; BMI:body mass index; BP: blood pressure; COPD: chronic obstructive pulmonary disease; CRT: cardiac resynchronization therapy; eGFR: estimated glomerular filtration rate; ICD: implantable cardioverter-defibrillator;

LVEF: left ventricular ejection fraction; MRA: mineralocorticoid receptor antagonist; N/A: not available; NT-proBNP: N-terminal pro-brain natriuretic peptide; NYHA: New York Heart Association; ST2: interleukin 1 receptor-like 1; hs-TnT: high sensitivity troponin T.

Supplementary table 2.Number and management of missing values in the study cohort for every risk calculator.

	Variable	Baseline missing values n (%)	Missing values at 12 months n (%)	Applied values at baseline*	Applied values at 12 months*
**MAGGIC-HF**	Creatinine, umol/L	0 (0)	8 (2.2)	-	99.9
	LVEF, %	0 (0)	3 (0.8)	-	48
**SHFM**	Sodium, mmol/L	2 (0.5)	10 (2.8)	138	140
	Cholesterol, mg/dL	158 (44.3)	132 (37.0)	163	165
	Uric acid, mg/dL	174 (48.7)	31 (8.7)	7.3	7.1
	Hemoglobin, g/dL	0 (0)	12 (3.4)	-	13.4
	Lymphocytes, %	21 (5.9)	9 (2.5)	22	24
**BCN Bio-HF**	Sodium, mmol/L	2 (0.5)	10 (2.8)	138	140
	eGFR, ml/min/1.73m2	0 (0)	8 (2.2)	-	62.4
	Hemoglobin, g/dL	0 (0)	12 (3.4)	-	13.4

*For missing drug or device variables, they were assigned no drug/device.

eGFR: estimated glomerular filtration rate; LVEF: left ventricular ejection fraction.

Supplementary Figure 1. Violin plots of 1- and 3-year risk prediction with the three calculators. A) 1-year risk prediction. B) 3-year risk prediction. Red, baseline risk; green, risk at 12 months.

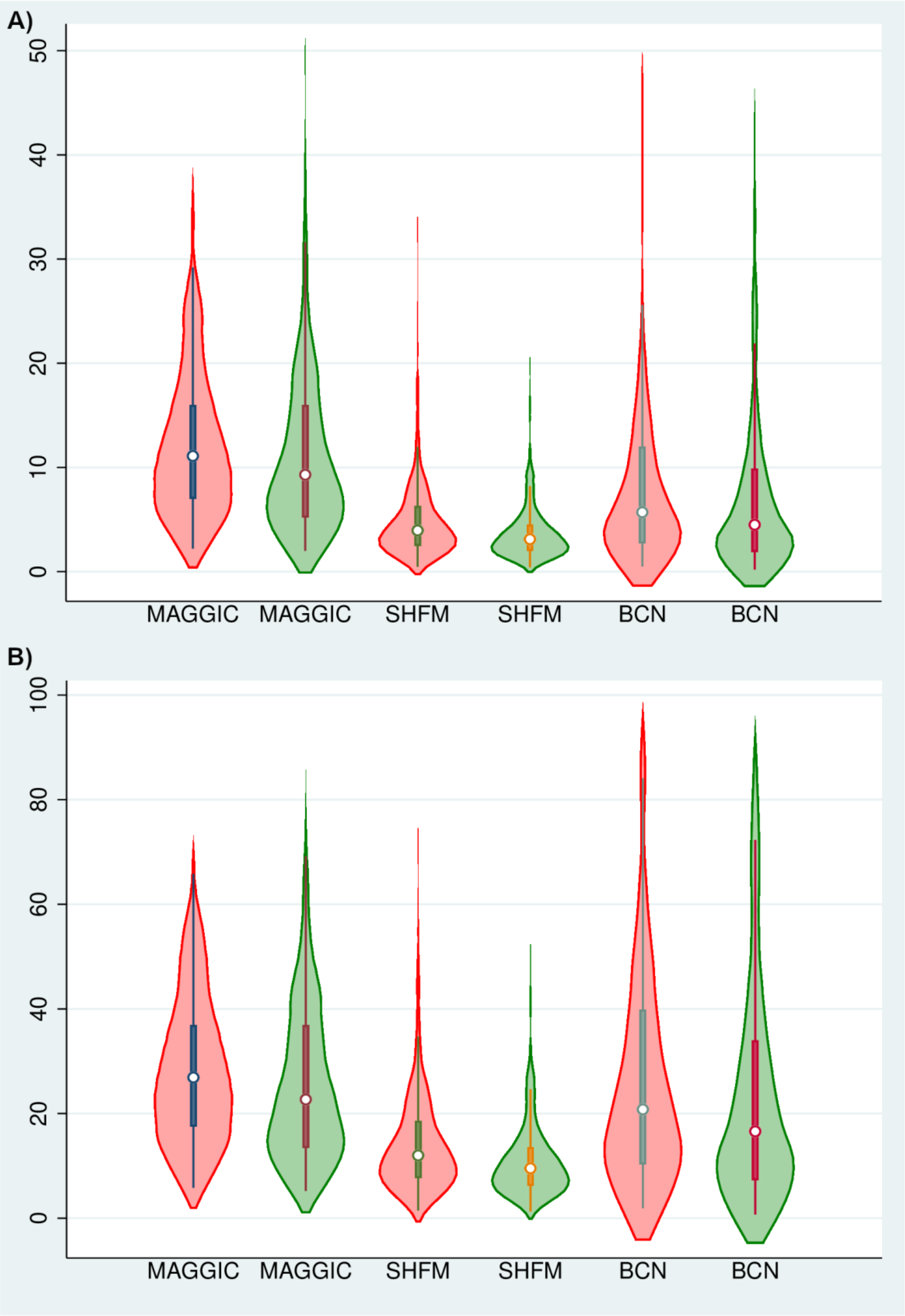

Supplementary Figure 2. Correlation between calculators in the absolute change of all-1-year cause death risk after 12 months of follow-up.

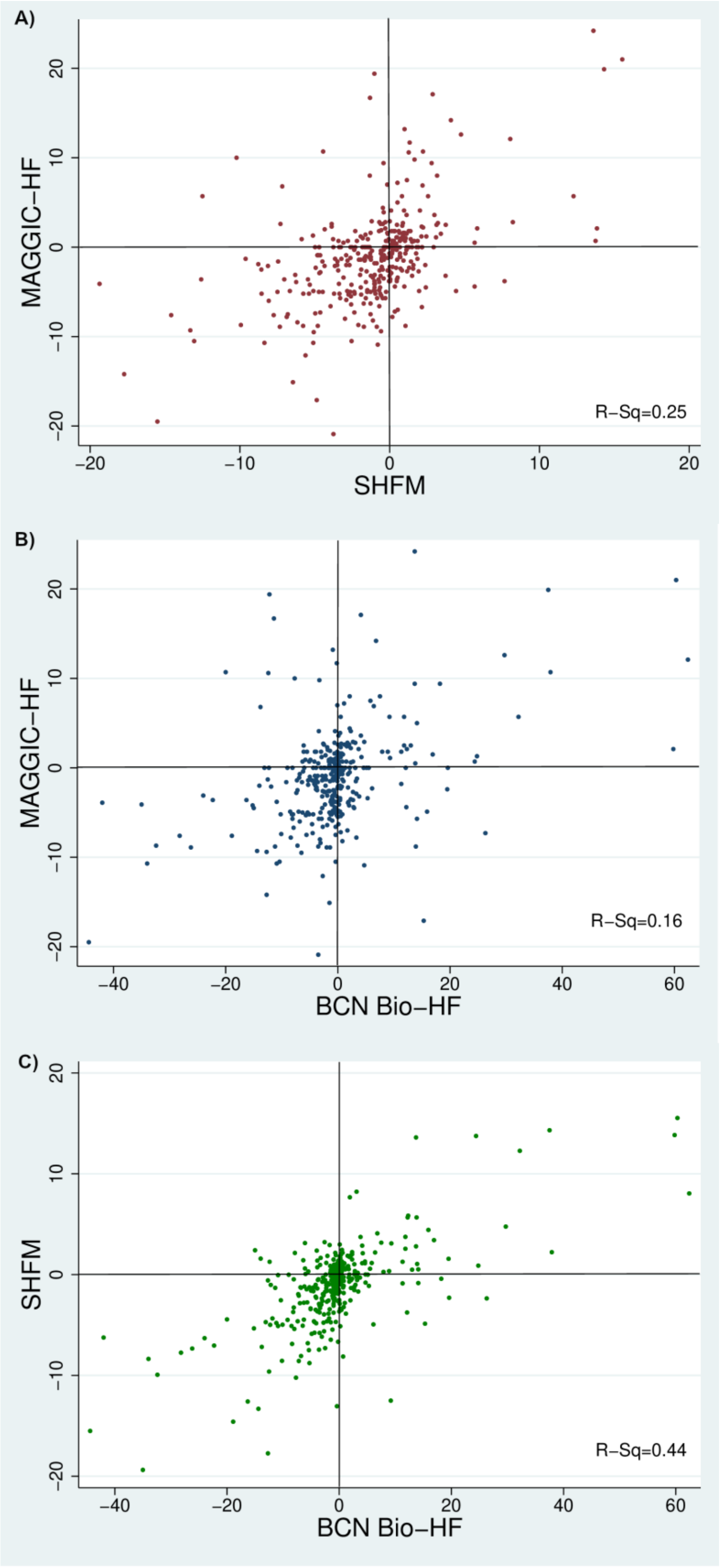

Supplementary Figure 3. Correlation within each calculator in the 1-year all-cause death risk at baseline and after 12 months of heart failure management.

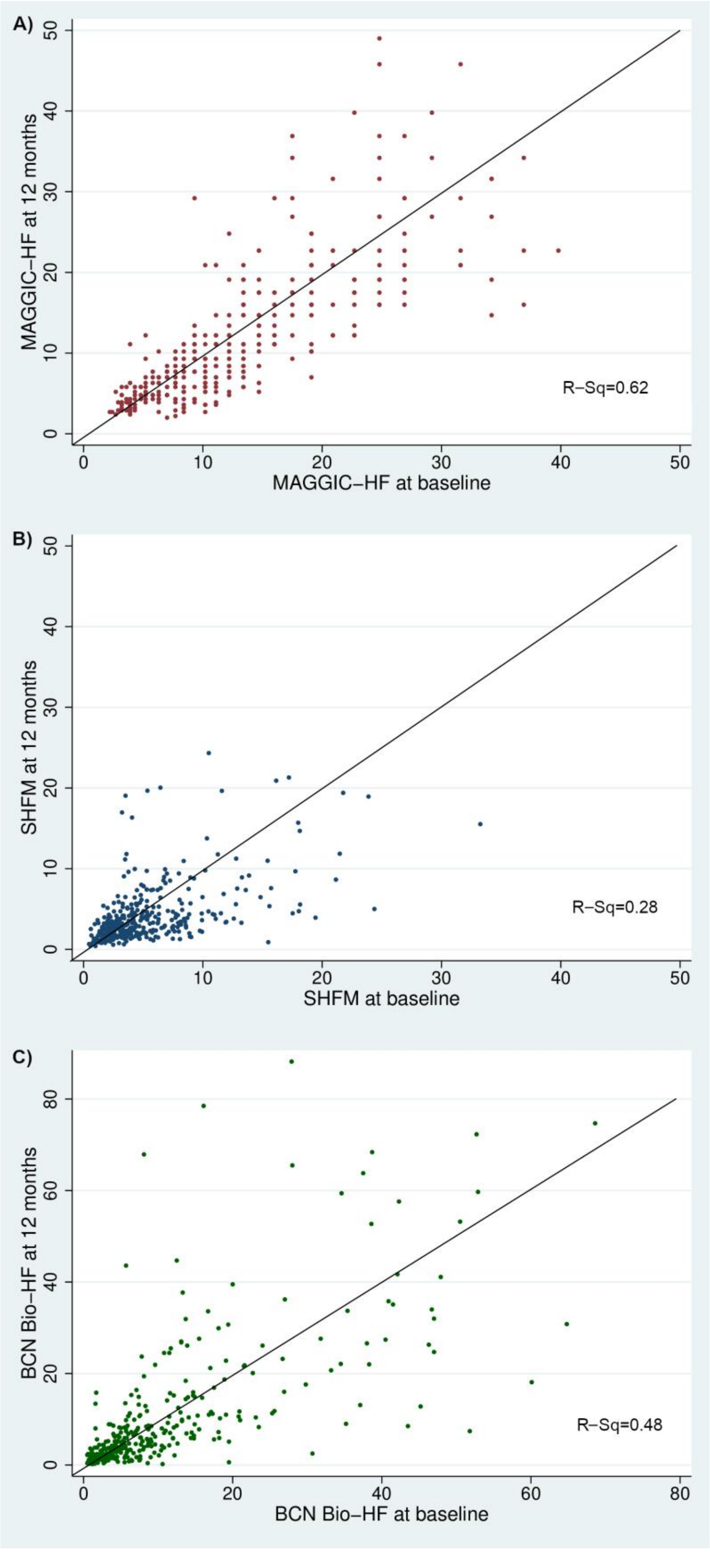

Supplement: Supplementary file 1 [file Data_Sheet_1.pdf]
